# A Foreign Body in the Eyeball Removed by Means of a Magnet

**Published:** 1886-04-20

**Authors:** A. G. Hobbs

**Affiliations:** Professor of Eye, Ear and Throat in the Southern Medical College, Atlanta, Ga.


### A FOREIGN BODY IN THE EYEBALL REMOVED BY MEANS OF A MAGNET.

BY A. G. HOBBS, M. D.,
**Professor of Eye, Ear and Throat in the Southern Medical College, Atlanta, Ga.**

Mr. A. W——, of Alabama, aged forty-two, while drilling with a steel drill, received a wound in the right eye, which gave him but little pain at first, but after twenty-four hours the pain became severe. His physician, Dr. A. B. Coleman, saw him on the evening of the day the injury occurred, and was sure he could see the piece of steel (looking at it, according to the patient's account, with an ophthalmoscope), but would not attempt to extract it The eye was at once atropenized, but, on the third day, the pain, photophobia and congestion had increased so much that the doctor referred him to me. I saw him on the fifth day, when I found a small opacity at the sclero-corneal junction, the point of entrance of the piece of steel. I also found considerable iritis, with inflammatory effusions, in the ciliary region. The effusion was so great that his vision was reduced to "finger counting," and it was impossible for me to see beyond the iris. Knowing that the good eye was in danger of becoming involved, through sympathy, I determined to try the magnet, though I had but little idea of the locality of the foreign body.

After thoroughly cocainizing the eye, I introduced a Græfe's knife through the cornea, and ciliary region, into the virtrious, following the course of the steel as near as possible, and just missing the lens. After the hemorrhage had ceased, I gently introduced the probe of a Gruning's magnet, and slowly withdrew it till, upon the fifth withdrawal, a small, irregular piece of steel, about half the size of the head of a pin, came out adhering to the point of the probe. There was but little loss of vitreous, a very small bead only following the fourth and fifth withdrawals of the probe. The eye was at once firmly bandaged, the patient placed at rest, and the case treated as an ordinary traumatism of the ciliary region.

The pain and inflammation gradually subsided under treatment, and the inflammatory exudations were slowly absorbed till his vision, at the end of three weeks, was $\frac{6}{xx}$. Two weeks later vision remained the same, but all symptoms had subsided, except a small band of iritis adhesions in the region of the wound, which left the pupil irregular in shape. I have no doubt that, had the magnet not been successfully used in this case, an enucleation would have been necessary. I expected that a traumatic cataract would be the result, but I have not heard that such has been the case. I infer that the vision still remains as good, if it has not improved, since the acute symptoms passed away.

